# No gesture too small: An investigation into the ability of gestural information to mislead eyewitness accounts by 5- to 8-year-olds

**DOI:** 10.3758/s13421-023-01396-5

**Published:** 2023-03-30

**Authors:** Kirsty L. Johnstone, Mark Blades, Chris Martin

**Affiliations:** Department of Psychology, Cathedral Court, The University of Sheffield, 1 Vicar Lane, Sheffield, S1 2LT UK

**Keywords:** Gesture, Eyewitness, Misinformation, False memory, Non-verbal communication, Children

## Abstract

The accuracy of eyewitness interviews has legal and clinical implications within the criminal justice system. Leading verbal suggestions have been shown to give rise to false memories and inaccurate testimonies in children, but only a small body of research exists regarding non-verbal communication. The present study examined whether 5- to 8-year-olds in the UK could be misled about their memory of an event through exposure to leading gestural information, which suggested an incorrect response, using a variety of question and gesture types. Results showed that leading gestures significantly corrupted participants’ memory compared to the control group (MD = 0.60, *p* < 0.001), with participants being misled by at least one question nearly three-quarters of the time. Questions about peripheral details, and gestures that were more visible and expressive, increased false memory further, with even subtle gestures demonstrating a strong misleading influence. We discuss the implications of these findings for the guidelines governing eyewitness interviews.

## Introduction

**Fig. 1:**
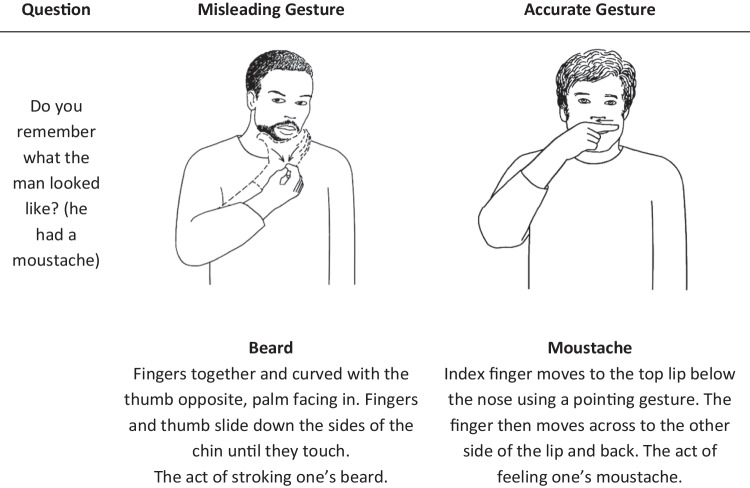
An example of a misleading and accurate gesture in the context of the question asked. Adapted from Simplified Signs: A Manual Sign-Communication System for Special Populations, 2020, 10.11647/OBP.0220 CC by 4.0

The fact that children are to some extent susceptible to misleading information has important legal and forensic implications for eyewitness testimony (Klemfuss & Olaguez, 2018). To obtain the best evidence possible, police interview guidelines include recommendations to reduce the possibility of *verbally* leading a witness (Ministry of Justice, 2022). No such guidelines exist regarding *non-verbal* communication, however, despite research showing that gestures can be as misleading as verbal information, a phenomenon known as the Gestural Misinformation Effect (GME) (Broaders & Goldin-Meadow, 2010; Gurney et al., 2013; Gurney et al., 2016; Kirk et al., 2015). For example, when children are asked if they remember what a man looked like, exposure to the misleading gesture of *beard* may lead them to believe the man did indeed have a beard (Fig. 1). The present study assesses the strength of the GME with the aim of informing current eyewitness interview practices. The study also addresses some methodological limitations of previous work in the area and includes examination of the effect of question centrality and gesture saliency, on the ability of gestures to mislead child witnesses.

Gestures are closely linked to speech, whether for the benefit of the speaker through helping to conceptualise and schematise information (Kita et al., 2017) or for the listener by enhancing understanding when speech is unclear or difficult to articulate (Dargue et al., 2019; Hostetter, 2011). Observing gestures has also been shown to improve memory through exploitation of the listener’s motor system to create stronger mental representations than speech alone (Ianì & Bucciarelli, 2017), while the activation of the frontal motor system upon gesture perception has been linked to prior experience of gesture production (Wakefield et al., 2013). Speech and gesture can be unintentionally combined into a single unified memory event despite differing information sources (Stark et al., 2010). For example, did that person say they did not know the answer, or did they shrug, and I interpreted it that way? Co-speech gestures have been shown to activate the same neurological networks as speech (Yang et al., 2015) during both observation as a listener (Xu et al., 2009) and production as the speaker (Marstaller & Burianová, 2015). This neurological link between speech and gesture means that even when information is reactivated during recall in the original modality (such as auditory or visual), this difference is not always available during recollection as a basis for source judgements (Mitchell & Johnson, 2009) or true/false memory decisions (Stark et al., 2010).

Evidence from the *verbal* misinformation literature (see Loftus, 2005, for review) often shows greater susceptibility in younger children to false memories from *suggestive* pressure such as repeated questioning, leading questions, and post event misinformation (Bruck & Ceci, 1999; Poole & Dickinson, 2014; Volpini et al., 2016). After a crime, exposure to post-event information can be particularly problematic for reliable evidence collection due to the inability to control a witness’s experiences prior to interview, such as television reports, social media, parental involvement, social workers, etc. If this post-event information is misleading (i.e., fabricated or inaccurate), it can give rise to false memories that are perceived to be true by the witness, and leading to potentially contaminated evidence (Loftus & Hoffman, 1989). The inclusion of post-event misinformation into a witness’s original memory is known as the *misinformation effect* (Loftus, 2005). Typical misinformation paradigms examining the misinformation effect characteristically include three phases: a witnessed event (e.g., a short vignette), followed by post-event information containing false details (e.g., the presence of a weapon), and finally an interview to assess the presence of false information in participants’ memory of the event.

Certain groups are more susceptible to the *verbal* misinformation effect including younger children (Gudjonsson et al., 2016; Volpini et al., 2016), the elderly (Biondi et al., 2020), and those with cognitive deficits (Morrison et al., 2019), indicating a developmental role of cognition in suggestibility. This is often thought to be due to improvements in cognitive processes from young children to older children and adults (Bruck & Melnyk, 2004) and/or greater resistance to social conformity pressures, such as trying to provide the ‘right’ answer over and above the original memory (Gudjonsson et al., 2016; Roebers et al., 2005). The idea that suggestibility reduces with age is relevant in a legal context where children are seen as inferior witnesses to adults (Bruer & Pozzulo, 2014). Mixed results have been seen regarding gender and suggestibility (Bruck & Melnyk, 2004), with stronger narrative skills and expressive language ability often seen in girls (Buckner & Fivush, 1998), who paradoxically show both better recall (Perez et al., 2022), *and* increases in suggestibility (Kulkofsky & Klemfuss, 2008). Advanced narrative ability can also predict reduced recognition accuracy in girls, but not boys (Klemfuss & Wang, 2017), indicating an interesting relationship between gender and accuracy.

Under conditions in which true and false information is semantically connected (e.g., experiencing a robbery and later recalling false information such as the presence of a weapon), developmental reversals may be seen, with older children and adults showing greater susceptibility to false memory than children (Howe et al., 2009). This is supported by the associative-activation theory (AAT) (Wimmer & Howe, 2009), which states that false memory is a result of the spreading activation of associated words and concepts contained in an individual’s knowledge base developing into a meaningfully related, but false, spontaneous memory. As such, the larger knowledge base available, and the faster and more automatic way in which information is accessed in older children and adults (Otgaar et al., 2018), can result in higher rates of misinformation acceptance with age. Individual differences in familiarity and experience can help explain variations in spontaneous false memory formation in children, with higher gist-trace processing (the level of processing of the associative semantic content in a memory) predicting false memory formation above that of chronological age (Bouwmeester & Verkoeijen, 2010; Brainerd et al., 2008). Differing findings for suggested and spontaneous false memories challenge the view that children are unreliable witnesses and highlight how predications regarding the reliability of an eyewitness should not be based on age alone.

The ability of misleading gestures to disrupt memory recall is known as the gestural misinformation effect (GME) (Kirk et al., 2015). The GME has been demonstrated in adults (Gurney et al., 2016) and children (Kirk et al., 2015), with an open question including a misleading gesture showing the same ability to mislead children as a verbally misleading question (Broaders & Goldin-Meadow, 2010). Gestural information can also mislead in conditions that would usually protect children against verbal suggestions such as higher verbal ability, older age, or increased memory trace strength (Kirk et al., 2015). Kirk et al. (2015) found that children were misled by at least one misinformation detail 25 out of a possible 30 times, with some children additionally mirroring the gestures used by the interviewer. With evidence of enhanced learning and memory of new concepts in children when speech and gesture are combined (Cook et al., 2008), Kirk et al. (2015) reasoned that the encoding of gestural misinformation may be a more powerful route through which false memories are formed than speech alone.

At present, research regarding the gestural misinformation effect in children is limited despite evidence showing that most interviewers use meaningful gestures to some degree, and that gesture use increases when interviewing younger children (Meyer, 2019). Additionally, despite a number of studies examining how *verbal* suggestibility changes with age, the effect of age on *gesture* suggestibility has not been adequately investigated. Previous methodologies need to be expanded to consider how different types of questions and gestures might interact with an accurate or misleading condition to make findings more robust. Prior methodological limitations include questions being asked only in either a misleading or an accurate condition, but not both (Broaders & Goldin-Meadow, 2010), and between-participant designs resulting in participants only being exposed to one condition (accurate or misleading), and with no baseline control measure (Kirk et al., 2015). Understanding how question type and gesture type interact is a key consideration for police interviews. For example, questions that are more central to the event in question may create a stronger memory trace due to the higher level of perceptual detail involved and thus be better protected against misleading information (Migueles & Garcia-Bajos, 1999; Sarwar et al., 2014), while more expressive gestures may increase the ability of the gesture to cue the original memory trace, resulting in greater accuracy, or indeed a greater ability to mislead, if the gesture is utilised as a source of information above that of the original memory (Chu et al., 2014; Lindsay & Johnson, 2000).

In forensic contexts, information is classed as central or peripheral depending on its closeness to the ‘plot’ or forensic event in question, with central information defined as details related to the central characters and action, and peripheral information as non-central characters/action and details that occurred before or after the event (Ibabe & Sporer, 2004). Children typically show better integration and recall for the main action/plot of an event than peripheral details (Migueles & Garcia-Bajos, 1999; Sarwar et al., 2014), potentially making some questions easier to answer and less vulnerable to misleading gesture effects than other questions. Gesture saliency has not been considered in previous research, despite evidence that more salient gestures (more expressive and visible) are attended to more by the listener (Chu et al., 2014). Nor is it known to what extent more subtle gestures may affect false memory, and it is therefore unclear whether any level of gesture is safe during an interview. For example, some gestures may be more influential due to increased neurological engagement (Ianì & Bucciarelli, 2017; Yang et al., 2015), or as a source of information when the memory trace is weak for a particular answer, or if a question is ambiguous (Dargue et al., 2019; Pezdek & Roe, 1995).

An understanding of the role of question and gesture types in leading questioning is important to meaningfully inform future interview practice. Police interviewers are currently taught to use neutral mannerisms/speech during feedback so as to avoid confirming or agreeing with witness reports, for example, nodding their head/expressing surprise (College of Policing, 2022). There are no official guidelines, however, regarding the ability of gesture to lead the primary evidence of a witness, leaving testimony vulnerable to potentially misleading information that may reduce the chances of a case proceeding to prosecution. To address key gaps in research, the present study assessed the strength of the gestural misinformation effect when questions were asked not only about central events and characters, but also about peripheral details. A variety of gestures were used, which included a mix of gestures that were salient and highly visible (e.g., whole arm movements), and gestures that were subtle and less obvious to the listener (e.g., naturalistic hand or finger movements) (Chu et al., 2014). In the present study, all the questions were counterbalanced and subjected to appropriate control conditions (accurate, misleading, and no gesture) so that each child was exposed to each question type (central and peripheral) and each gesture condition (salient and subtle).

Previous researchers have shown that biological and cognitive changes in memory between 6 and 7 years of age are associated with better recall of events (Fritz et al., 2010; Geurten & Willems, 2016; Ghetti, 2003). Based on this evidence, and research indicating that verbal suggestibility varies as a function of age (Bruck & Ceci, 1999; Gudjonsson et al., 2016; Volpini et al., 2016), two age groups were tested (5- to 6-year-olds and 7- to 8-year-olds) using a mixed method design with age as the between participant variable and condition (type of gesture and question) as within-participant variables. It was hypothesised that the gestural misinformation effect would decrease with age due to improvements in memory and recall resulting in less reliance on gesture as a source of information. Given the relative closeness of the ages being tested, it was not thought that increased semantic connections in the 7- to 8-year-olds would be sufficient to reverse developmental trends within the study, and that any meaningful information aiding gist-trace processing, or associative activation, would be the same for both age groups.

It was expected that *accurate* gestures would lead to more correct responses than *no gesture,* due to their ability to cue the original memory (Lindsay & Johnson, 2000), and that misleading gestures would lead to more incorrect answers than *no gesture* due to the mismatch with the original memory and the gesture being used as a basis for information (Dargue et al., 2019; Pezdek & Roe, 1995). Following Kirk et al. (2015) and Broaders and Goldin-Meadow (2010), it was hypothesised that a significant portion of incorrect answers would be consistent with the misleading gesture due to source misattribution (Brubacher et al., 2019), suggestive pressure (Bruck & Ceci, 1999; Poole & Dickinson, 2014; Volpini et al., 2016), or utilisation of the gesture as a source of information (Dargue et al., 2019; Pezdek & Roe, 1995). Although not directly tested before, salient gestures were expected to provide stronger visual cues than subtle gestures, resulting in more participants being misled. The stronger memory traces associated with central events over peripheral events (Migueles & Garcia-Bajos, 1999; Sarwar et al., 2014) were expected to protect against misleading gestures, resulting in less incorrect answers. Post-interview free recall was hypothesised to include more items of information (IOIs) than pre-interview free recall, including gestural information that participants had been exposed to during the interview, as observed by Kirk et al. (2015) and by Broaders and Goldin-Meadow (2010).

## Method

### Participants

A power analysis was conducted with G Power, for a mixed-model ANOVA with two groups and three measures, with a power of 0.80, a significance effect of α = 0.05, and a small to medium effect size η^2^ = 0.06 (f = 0.25). This resulted in a suggested sample size of 28. A small to medium effect size was deemed suitable due to research into the smallest effect size of interest (SESOI) regarding misinformation paradigms, and expert opinions that even a small effect is of psychological significance because of the possible negative consequences of even one incidence of false memory in forensic contexts (Riesthuis et al., 2022). Due to the availability of participants, and a reluctance to exclude children who wanted to participate and met the inclusion criteria, a larger sample size was used. As a result, a total of 63 primary school children were recruited aged 5–6 years (*n* = 31, 9 boys, 22 girls, *M* = 5.77 years, *SD* = 0.43) and 7–8 years (*n* = 32, 19 boys, 13 girls, *M* = 7.66 years, *SD* = 0.48). Sixty-five children were initially recruited, but two had special needs which affected their ability to attend to the video and the questions, and these children were excluded from the study. Children were recruited through a school in the UK as a sample of convenience and were predominantly of a white British background. Ethics approval for this study was given by the University of Sheffield. ﻿

### Materials

The children were asked to watch a 5-min video of a young girl taking a gymnastics examination. The video showed the girl doing gymnastics on a beam and included scenes of her practising when she was younger. There was little dialogue in the video. The video was presented on an iPad and the children’s responses were recorded using a digital recorder.

Three scripts were produced with 12 questions each containing four *no gesture* questions, four *accurate* gesture conditions, and four *misleading* gesture conditions (see Online Supplementary Materials (OSM), Table 1). All children received four questions from each condition and were not assigned to one condition only. Each script was counterbalanced so that (for example) a question asked in script 1 with no gesture, was asked with an accurate gesture in script 2, and a misleading gesture in script 3. Six questions on each script were based on central information, and six on peripheral information. This was the same for gesture saliency, with six questions including gestures that were more salient and six that were more subtle. This led to each question being categorised either as central/salient (×3), central/subtle (×3), peripheral/salient (×3), or peripheral/subtle (×3) in each script.

**Table 1:** The three question sets used with a description of each gesture

Questions	Centrality/Saliency	Set 1	Set 2	Set 3
Do you remember what Molly did to get the chalk off her hands before she started her exam?	Central/Salient	No gesture - Interviewer placed her hands, still, on table	Misleading - Interviewer rubbed hands repeatedly on chest	Accurate - Interviewer clapped hands gently in front of her
What about Molly's leotard, it was white and another colour, do you remember which colour?	Central/Subtle	Accurate -Interviewer indicated a red folder on the table	No gesture - Interviewer placed her hands, still, on table	Misleading - Interviewer indicated a blue folder on the table
Can you describe what the judge sitting on the left of the screen (indicate left) looked like?	Peripheral/Subtle	Misleading - Interviewer stroked chin with thumb and finger to suggest a beard	Accurate -Interviewer touched her top lip with her finger to suggest a moustache	No gesture - Interviewer placed her hands, still, on table
What about the judge on the right (indicate right), could you describe him for me?	Peripheral/Salient	No gesture -Interviewer placed her hands, still, on table	Misleading - Interviewer raised both hands just above her head and moved hands down to suggest the action of putting on a hat	Accurate - Interviewer circled fingers round eyes to suggest glasses
When Molly finished her exam how did her friend congratulate her?	Central/Salient	Accurate - Interviewer wrapped her arms around her body to suggest a hug	No gesture - Interviewer placed her hands, still, on table	Misleading - Interviewer moved hand as if giving someone a high five
Molly’s coach also came to speak to her when she had finished, do you remember how long the sleeves were on her coach’s top?	Peripheral/Salient	Misleading - Interviewer drew a line on her wrist with her hand to suggest long	Accurate - Interviewer drew a line on her arm just below her shoulder to suggest short	No gesture - Interviewer placed her hands, still, on table
At the end of the video how many girls were sat on the bench?	Central/Subtle	No gesture - Interviewer placed her hands, still, on table	Misleading - Interviewer held up three fingers low down casually while talking	Accurate - Interviewer held up five fingers low down casually while talking
Can you tell me what the judge did to let Molly know she could start the exam?	Peripheral/Salient	Accurate - Interviewer raised arm into the air	No gesture - Interviewer placed her hands, still, on table	Misleading - Interviewer did a thumbs up gesture
Do you remember what Molly's mum's hair looked like?	Peripheral/Subtle	Misleading - Interviewer indicated her own straight hair by smoothing her fingers down the strands	Accurate - Interviewer moved a finger in a spiral motion near her hair to indicate curly	No gesture - Interviewer placed her hands, still, on table
What about Molly's sister, do you remember what pattern was on her dress?	Peripheral/Subtle	No gesture - Interviewer placed her hands, still, on table	Misleading - Interviewer drew lines down her own top subtly to suggest stripes	Accurate - Interviewer tapped her finger in random places low down on her own top to suggest spots
Do you remember how Molly wore her hair during the exam?	Central/Subtle	Accurate - Interviewer picked up her own hair as though putting it in a hair band	No gesture - Interviewer placed her hands, still, on table	Misleading - Interviewer indicated her own hair which was loose
How do think Molly felt when she kept falling off during practise?	Central/Salient	Misleading - Interviewer made an angry face and shook her fist	Accurate - Interviewer pulled a sad face and trailed finger from eye to suggest tears	No gesture - Interviewer placed her hands, still, on table

### Design

The experiment consisted of a 3 (Condition: No gesture, Accurate and Misleading) × 2 (Age: 5–6 and 7–8 years old) mixed design, with *condition* as the within-participant variable (*n* = 63), and *age* as the between-participant variable (*n* = 31 and 32). Incorrect responses were further analysed in relation to the misleading gesture given using a 2 (Answer: consistent, inconsistent) × 2 (Age: 5–6 and 7–8 years old) mixed design, with *answer* as the within-participant variable and *age* as the between-participant variable.

### Coding

Free-recall interviews were coded by items of information (IOIs), which were defined as any information the child gave about the video. For example, a child who said, “A girl was doing gymnastics when she was younger and got a bit older, and she kept trying, there were girls with blonde hair and orange hair, some had brown” was coded as having seven IOIs due to information about the main character (1 – girl), what she was doing (2 – gymnastics), what happened in the video (3 – she got older), the theme of the video (4 – she kept trying), and what some of the people looked like (5– blonde hair, 6 – orange hair, and 7 – brown hair).

IOIs were analysed to determine which ones were correct and incorrect for pre- and post-free-recall interviews, and then compared to see if participants gave new IOIs after the structured questions. New IOIs were coded as correct or incorrect, by the first author, and examined to see if any were consistent with the gestures to which participants had been exposed. Inter-rater reliability was determined for 40% of the sample, which was coded by an experienced researcher who was not otherwise involved in the study. There was good agreement between the two coders for the pre-interview free-recall, Kappa = 0.62, *p* < 0.001, and for the post-interview free-recall, Kappa = 0.86, *p* < 0.001.

Responses to the 12 interview questions were coded as correct, incorrect, or do not know/do not remember (DK/DR). DK/DR answers included non-verbal communication such as shaking their head to indicate ‘no’ or shrugging their shoulders. Incorrect responses to questions with a misleading gesture were further categorised into responses consistent with the misleading gesture, or responses inconsistent with the misleading gesture. Inter-rater reliability was determined for 20% of the participants, with analysis showing nearly complete agreement between raters, Kappa = 0.99, *p* < 0.001.

### Procedure

**Fig. 2:**
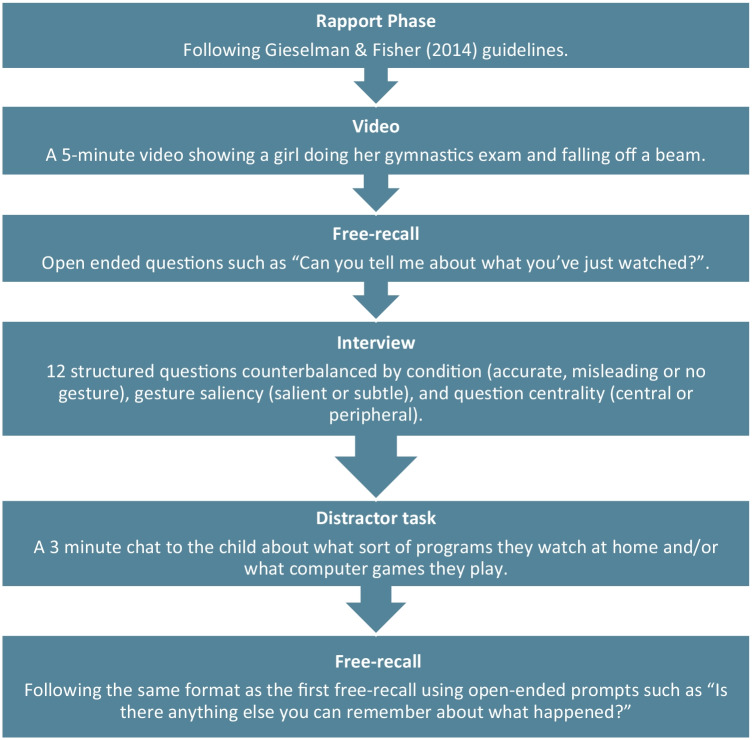
The interview procedure including a rapport phase and five stages *–* Video, Pre-interview free recall, Interview, Distractor task, and Post-interview free recall

Children were tested individually in a quiet area of the school and were seated next to the experimenter. The study began with a rapport phase and was then completed in five stages – watching the video, a pre-interview free recall, interview questions, a distractor task, and a post-interview free recall (Fig. 2).

All the children completed all stages. As per the enhanced cognitive interview guidelines (Fisher & Geiselman, 2010; Geiselman & Fisher, 2014), a rapport phase was included. Perceived control was handed from the interviewer to the child through statements such as “I’ve forgotten most of that video so do you think you could help me by answering some questions?”. Children were reassured that it was okay if they did not remember, or did not know an answer, as per police interview guidelines (Geiselman & Fisher, 2014). If the children did not pay attention or see the gesture, the interviewer got their attention verbally and the question was repeated once more. Children who were not able to attend to the video or the gestures (*n* = 2), still took part in the interview and received a certificate, but were later removed from analysis.

After the video each child was given the first free recall test and asked if they could tell the experimenter, in as much detail as possible, about the video that they had just watched. The same open-ended prompts were used to help elicit information from each child including ‘Can you remember anything else?’, ‘Do you know what happened next?’, and ‘Can you tell me a bit about any of the people in it?’.

After the first free recall interview, each child was randomly allocated one of three scripts and asked 12 structured questions (see Table 1 in the supplementary materials) across each of the three conditions (4 no gesture, 4 accurate and 4 misleading). In Condition 1 a question was asked with no gesture, so the experimenter kept her hands still on the table. In Condition 2 a question was asked with an accurate gesture, so the experimenter gave a gesture consistent with the information in the video. In Condition 3 a question was asked with a misleading gesture, so the experimenter gave a gesture inconsistent with information in the video. All gestures given were iconic gestures (McNeill, 1992), that is they formed a meaningful representation of a concept visually, e.g., miming putting on a hat to represent ‘hat’, and were chosen so as to convey semantic information to the participant.

To examine whether the centrality of the event affected the level to which participants could be misled by gesture, six questions were about the central character and/or action, and six questions were about peripheral details. The centrality of a question was determined by its closeness to the event in question. Central questions were about the main character and the action sequence that happened, whereas peripheral questions focussed on events that took place before or after the main event, or were about other people in the video (Andrews & Lamb, 2019). Gestures were split evenly into subtle or salient gestures (Chu et al., 2014). Salient gestures required whole arm movements above the chest, while subtle gestures required movement involving just the hand or fingers.

After answering the questions, children were given a distractor task for three minutes, during which they talked about their favourite TV or film characters. Then the children were given a second free recall-test and were again asked if they could tell the experimenter as much information as possible about the video that they had watched. The same open-ended prompts were used as in the first recall test.

## Results

### Type of gesture and answer type

**Fig. 3:**
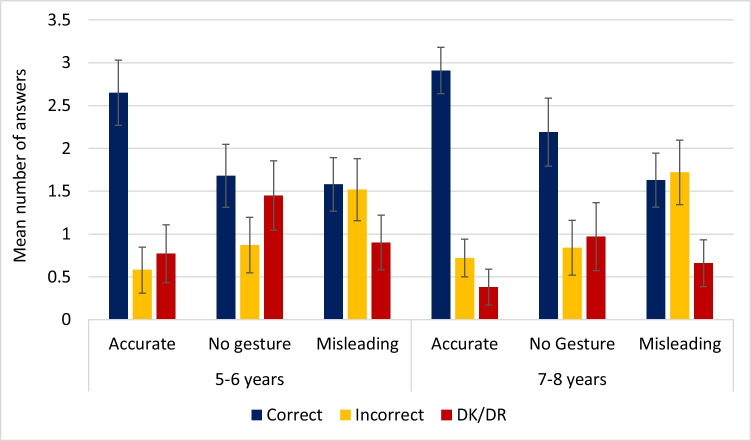
Mean number of correct, incorrect, and DK/DR responses (out of a maximum score of 4) for 5- to 6-year-olds and 7- to 8-year-olds, including 95% confidence intervals

The mean number of questions that children answered correctly, incorrectly, or were not able to answer, for each condition are given in Table 2 and visually represented in Fig. 3. Given the number of comparisons being made, all results were adjusted using Bonferroni’s correction to give a conservative significance estimate, unless otherwise stated.

**Table 2:** The mean and standard deviation of correct, incorrect and DK/DR responses to each of the three conditions (accurate, no gesture and misleading) for each age group

	Condition	Mean (SD)		
		Correct	Incorrect	DK/DR
5–6 years	Accurate	2.65 (1.08)	0.58 (0.76)	0.77 (0.96)
	No gesture	1.68 (1.05)	0.87 (0.92)	1.45 (1.15)
	Misleading	1.58 (0.89)	1.52 (1.03)	0.9 (0.91)
7–8 years	Accurate	2.91 (0.78)	0.72 (0.63)	0.38 (0.61)
	No Gesture	2.19 (1.15)	0.84 (0.92)	0.97 (1.15)
	Misleading	1.63 (0.91)	1.72 (1.08)	0.66 (0.79)

A 3 × 2 factorial ANOVA was conducted to test the within-participant effect of condition (no gesture, accurate gesture, or misleading gesture) and the between-participant effect of age (5–6 years and 7–8 years old) on correct responses.

A significant main effect of gesture type for correct responses was found, *F*(2,122) = 28.27, *p* < 0.001, *η*^*2*^ = 0.32. Pairwise comparisons with Bonferroni adjustment revealed that accurate gestures were significantly more likely to elicit a correct response compared to both the no-gesture condition (mean difference (*MD*) = 0.84, *p* < 0.001, Cohen’s *d* = 0.85) and the misleading gesture condition (*MD* = 1.17, *p* < 0.001, Cohen’s *d* = 1.19). A misleading gesture was less likely to elicit a correct response compared to the no-gesture condition (*MD* = 0.33, *p* = 0.125, Cohen’s *d* = 0.33). No main effect of age was found for correct responses *F*(1, 61) = 2.76, *p* = 0.102, *η*^*2*^ = 0.043, and there was no interaction between age and gesture for correct responses *F*(2,122) = 1.05, *p* = 0.353, *η*^*2*^ = 0.017.

A second 3 × 2 factorial ANOVA was conducted to test the within-participant effect of condition (no gesture, accurate gesture, or misleading gesture) and the between-participant effect of age (5–6 years and 7–8 years old) on incorrect responses. Results showed a significant main effect of gesture type on incorrect answers *F*(2,122) = 23.16, *p* < 0.001, *η*^*2*^ = 0.275, with pairwise comparisons showing misleading gestures were significantly more likely to produce an incorrect answer when compared to both the control condition (*MD* = 0.76, *p* < 0.001, Cohen’s *d* = 0.85) and the accurate condition (*MD* = 0.97, *p* < 0.001, Cohen’s *d* = 1.08), while no effect was seen on the number of incorrect answers between accurate and no gesture conditions (*MD* = 0.21, *p* = 0.418, Cohen’s *d* = 0.23). No main effect of age was found for incorrect responses *F*(1, 61) = 0.49, *p* = 0.485, *η*^*2*^ = 0.008, and there was no interaction between age and gesture for incorrect responses *F*(2,122) = 0.31, *p* = 0.731, *η*^*2*^ = 0.005.

### The ability of gesture to mislead

**Fig. 4:**
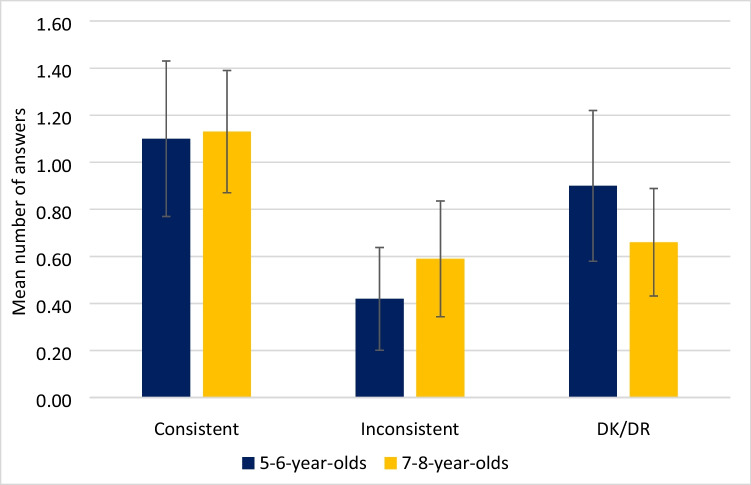
Mean number of incorrect responses that were consistent or inconsistent with the misleading gesture given, or DK/DR, including 95% confidence intervals

Incorrect responses were further analysed to examine whether the incorrect response was consistent with the misleading gesture given, inconsistent with the gesture given, or whether children did not know or could not remember (DK/DR) (Fig. 4). For example, when asked “what was the judge on the right wearing?” followed by the misleading gesture of hat, a response of ‘hat’ was classed as a consistent response, but a response of ‘coat’ was classed as an inconsistent response. All inconsistent answers were closely related to the question asked. For example, questions regarding appearance all elicited incorrect answers that ‘could’ have been correct such as a blue jacket, or a flowered dress, or pigtails. Questions regarding actions elicited action answers which could have occurred, but did not.

A significant main effect of response type *F*(1.8,110.7) = 7.11, *p* = 0.002, *η*^*2*^ = 0.104 was found, with incorrect answers *consistent* with the misleading gesture produced significantly more often (*MD* = 0.60, *p* < 0.001, Cohen’s *d* = 0.76) than incorrect answers *inconsistent* with gestures. No difference was found between inconsistent and DK/DR responses (*MD* = 0.27, *p* = 0.255, Cohen’s *d* = 0.34) or between consistent and DK/DR responses (*MD* = 0.33, *p* = 0.226, Cohen’s *d* = 0.42).

**Fig. 5:**
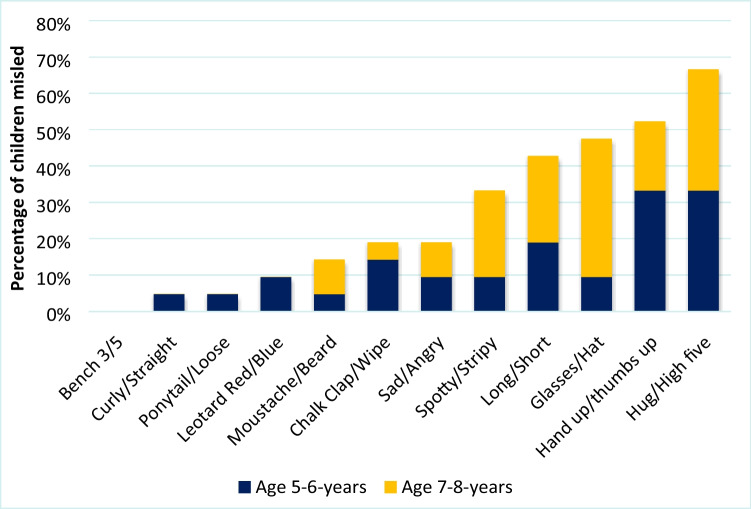
The combined percentage of children misled by the 12 possible gestures over the whole sample (*N* = 63). Each misleading question was asked 21 times in total and was answered by 21 different children

Out of the four misleading questions given to each participant, 70% of the 5- to 6-year-olds and 75% of the 7- to 8-year-olds were misled by at least one question (Fig. 5). Out of the 12 possible misleading questions, the misleading gesture that had the greatest effect overall was the hug versus high five gesture, with 66% of children overall giving a response consistent with the misleading gesture. The least misleading gesture was the gesture denoting how many children sat on the bench at the end of the video, with no children from either age group responding with ‘three’ as was suggested by the gesture.

### Pre- and post-free-recall analysis

To further examine the capability of gestural information to affect memory, five 2 × 2 factorial ANOVAs were conducted to test the effect of age on pre- and post-free-recall responses in relation to the number of words spoken, the number and proportion of correct IOIs, and the number and proportion of incorrect IOIs (Table 3). The proportions of IOIs were calculated by dividing the value of the part (correct or incorrect IOI) by the value of the whole (total IOIs).

**Table 3:** Mean and standard deviations of free recall answers pre- and post-interview, for each age group

	Age 5–6 (*n* = 31)		Age 7–8 (*n* = 32)	
	Pre-interview	Post-interview	Pre-interview	Post-interview
Total number of words	41.32 (45.41)	31.80 (34.04)	47.56 (50.82)	46.94 (43.93)
Correct IOIs	4.81 (3.62)	4.81 (4.50)	6.28 (3.42)	7.81 (7.09)
Incorrect IOIs	0.52 (1.81)	0.55 (0.89)	0.13 (0.34)	0.94 (1.70)
Proportion correct IOIs	0.82 (0.31)	0.65 (0.39)	0.86 (0.27)	0.74 (0.36)
Proportion incorrect IOIs	0.05 (0.12)	0.08 (0.13)	0.02 (0.05)	0.10 (0.16)

*IOI* (item of information)

A main effect of age was seen for the number of correct IOIs (*F(*1,61) = 4.59, *p* = 0.036, *η*^*2*^ = 0.070) with older children recalling 1.47 more correct IOIs pre-interview, and 3.00 more correct IOIs post- interview, than the younger sample. The *proportion* of correct and incorrect IOIs were examined to understand how much of the information given as a whole was correct/incorrect in comparison to other IOIs given by that participant. A significant change was seen pre- and post-interview for the *proportion* of correct IOIs (*F*(1,61) = 7.11, *p* = 0.010, *η*^2^ = 0.104) and the *proportion* of incorrect IOIs (*F*(1,61) = 6.83, *p* = 0.011, *η*^*2*^ = 0.101) with more IOIs for each found post-interview.

No effect of condition (pre- and post-interview) was found for the number of words spoken (*F*(1,61) = 0.78, *p* = 0.382, *η*^*2*^ = 0.013), the number of incorrect IOIs (*F*(1,61) = 3.01, *p* = 0.088, *η*^*2*^ = 0.047), or the number of correct IOIs (*F*(1,61) = 1.37, *p* = 0.246, *η* ^*2*^ =0.022). There were no age effects for the number of words spoken (*F*(1,61) = 1.27, *p* = 0.264, *η*^*2*^ = 0.02), the number of incorrect IOIs (*F*(1,61) = 0.00, *p* = 0.997, *η*^*2*^ = 0.00), the proportion of correct IOIs (*F*(1,61) = 1.03, *p* = 0.313, *η*^*2*^ = 0.017), or the proportion of incorrect IOIs (*F*(1,61) = 0.05, *p* = 0.817, *η*^*2*^ = 0.001).

### Centrality/saliency and the misinformation effect

To establish whether question centrality or gesture saliency affected the strength of the gestural misinformation effect, each question was examined for its level of centrality to the event in question, and for the saliency of the gesture used (Table 4). Peripheral details (*M* = 0.65) were more likely to elicit the suggested word than central events (*M* = 0.41), *t*(62) = 1.99, *p* = 0.050, Cohen’s *d* = 0.25. Of the top five misleading questions, four out of five questions asked about peripheral details, while central events showed a more varied response (see Table 4). Salient gestures (*M* = 0.83) were significantly more likely to elicit the misleading suggested word than gestures which were more subtle (*M* = 0.23), *t*(62) = 5.45, *p* < 0.001, Cohen’s *d* = 0.69. The top four misleading questions were those accompanied by salient gestures, with the most misleading question containing the largest whole arm movement above the chest, and were thus the most salient, while the five least misleading questions all contained more subtle naturalistic gestures (see Table 4).

**Table 4:** The number of children misled by each question and gesture type. Each question was asked a total of 21 times, to 21 different children

Question	Percent of children misled		Central/peripheral	Salient/subtle	Total misled out of 21	Percent total misled (*n* = 21)
	Age 5–6	Age 7–8				
Hug/High five	64%	70%	Central	Salient	14	67%
Raise hand/Thumbs up	64%	40%	Peripheral	Salient	11	52%
Glasses/Hat	20%	73%	Peripheral	Salient	10	48%
Long/Short	40%	45%	Peripheral	Salient	9	43%
Stripes/Spots	20%	45%	Peripheral	Subtle	7	33%
Angry/Sad	20%	18%	Central	Salient	4	19%
Clap/Wipe	30%	9%	Central	Salient	4	19%
Beard/moustache	10%	18%	Peripheral	Subtle	3	14%
Red/Blue	18%	10%	Central	Subtle	3	14%
Hair up/Down	9%	0	Central	Subtle	1	5%
Straight/Curly	10%	0	Peripheral	Subtle	1	5%
3 or 5	0	0	Central	Subtle	0	0%

### No gesture and DK/DR answers

**Fig. 6:**
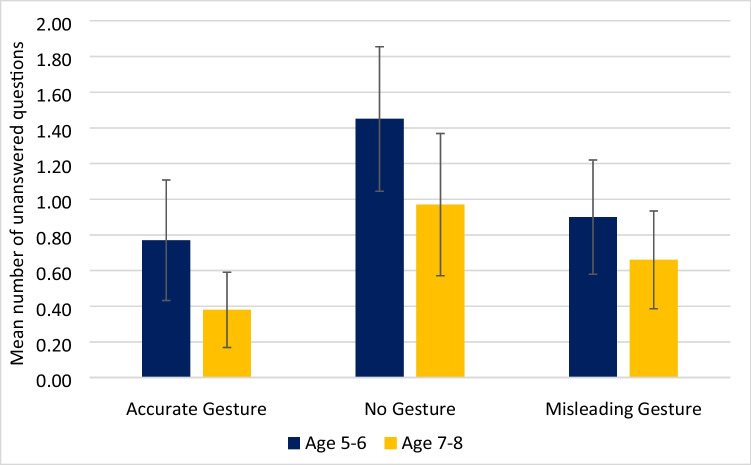
The mean number of DK/DR answers for each condition and age group, including confidence intervals

To assess if the condition a question was asked in (accurate, no gesture or misleading) affected the ability of children to give an answer, the number of DK/DR answers in each condition and age group was examined (Fig. 6). A significant main effect of condition was found *F*(2,122) = 9.98, *p* < 0.001, *η*^*2*^ = 0.14, with planned comparisons with Bonferroni correction showing that questions accompanied by no gesture were significantly more likely to elicit DK/DR answers than either an accurate gesture (*MD* = 0.64, *p* < 0.001, Cohen’s *d* = 0.67) or a misleading gesture (*MD* = 0.43, *p* = 0.020, Cohen’s *d* = 0.45).

The five questions most likely to elicit DK/DR answers were all questions regarding peripheral details, and the five questions least likely to elicit DK/DR answers were all central details. Questions classed as peripheral accounted for 77% of all DK/DR answers, compared to 23% for central events. The spread of DK/DR responses with regard to saliency showed 54% of questions produced a DK/DR answer when gestures were subtle, compared to 46% when gestures were salient. No discernible pattern could be seen in the effect of gesture saliency on DK/DR answers.

### Suggestibility as a function of age

Examining the effect of age on a gesture’s ability to mislead showed no main effect of age for correct responses *F*(1, 61) = 2.76, *p* = 0.102, *η*^*2*^ = 0.043, incorrect responses *F*(1, 61) = 0.49, *p* = 0.485, *η*^*2*^ = 0.008, or incorrect answers consistent with the misleading gesture *F*(1,61) = 0.04, *p* = 0.845, *η*^*2*^ = 0.001, indicating that the ability of gestural information to mislead was not related to the age of the participants.

There were no age differences between erroneous answers consistent with the suggested word for central *t*(61) = 0.95, *p* = 0.347, Cohen’s *d* = 0.24; or for peripheral events *t*(61) = 1.19, *p* = 0.240, Cohen’s *d* = 0.30; or salient *t*(61) = 0.207, *p* = 0.836, Cohen’s *d* = 0.05; or subtle gestures *t*(61) = 0.205, *p* = 0.839, Cohen’s *d* = 0.05, indicating a lack of developmental change. A significant age difference was seen between groups for DK/DR responses, with younger children providing DK/DR answers more often than older children *F*(1,61) = 4.95, *p* = 0.030, *η*^*2*^ = 0.08, but there was no interaction between age and condition *F*(2,122) = 0.34, *p* = 0.713, *η*^*2*^ = 0.01.

### Exploratory gender analysis

Given the difference in gender in the two age groups, exploratory analyses were conducted to look at the effect of gender on free recall, on correct/incorrect/DK/DR responses, and on gestural information’s ability to mislead, to see if this may have affected the lack of age effects seen. Five 2 × 2 factorial ANOVAs were completed to test the effect of gender (boys or girls) on pre- and post-free-recall responses in relation to the number of words spoken, the number and proportion of correct items of information (IOI), and the number and proportion of incorrect IOIs (Table 5).

**Table 5:** Mean (SD) of free recall measures pre and post by gender

	Boys (*n* = 28)		Girls (*n* = 35)	
	Pre-interview	Post-interview	Pre-interview	Post-interview
Total number of words	31.31 (23.02)	26.75 (35.74)	55.20 (59.20)	49.69 (40.40)
Correct IOIs	5.14 (3.60)	4.21 (5.12)	5.89 (3.56)	8.02 (6.35)
Incorrect IOIs	0.18 (0.39)	0.75 (1.76)	0.43 (1.70)	0.74 (0.98)
Proportion correct IOIs	0.81 (0.34)	0.56 (0.44)	0.86 (0.24)	0.80 (0.27)
Proportion incorrect IOIs	0.03 (0.07)	0.07 (0.13)	0.03 (0.10)	0.11 (0.17)

*IOI* item of information

An effect of gender was seen for the total number of words spoken (*F*(1,61) = 6.59, *p* = 0.013, *η*^*2*^ = 0.10) with girls giving a mean of 23.51 more words than boys, for the number of correct IOIs (*F*(1,61) = 4.70, *p* = 0.034, *η*^*2*^ = 0.07) with girls giving a mean of 2.28 more correct IOIs than boys, and for the proportion of correct IOIs *F*(1,61) = 5.92, *p* = 0.018, *η*^*2*^ = 0.09) with girls showing a higher proportion of correct IOIs (*MD* = 0.15) than boys. No effect of gender was seen for the number of incorrect IOIs *F*(1,61) = 0.28, *p* = 0.599, *η*^*2*^ = 0.01), or for the proportion of incorrect IOIs *F*(1,61) = 0.95, *p* = 0.333, *η*^*2*^ = 0.02). This indicates that during free recall, girls gave more correct information than boys, but boys and girls were equally likely to give incorrect information.

**Fig. 7:**
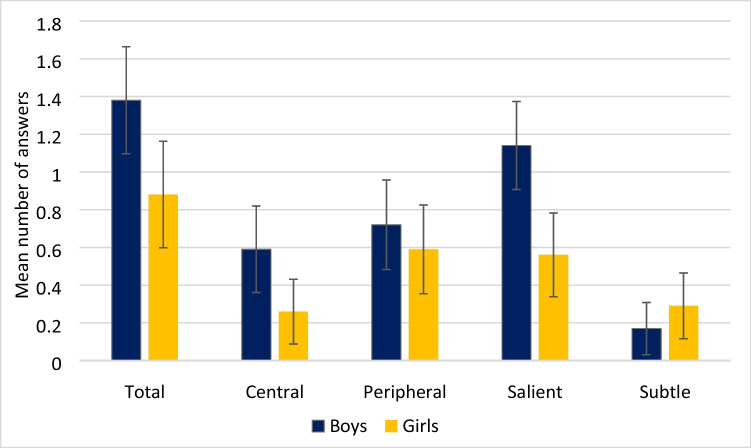
The mean number of answers consistent with the misleading gesture given, including confidence intervals, by gender

No differences were seen for gender with regard to the number of correct (*F*(1,61) = 1.31, *p* = 0.257, *η*^*2*^ = 0.02), incorrect (*F*(1,61) = 0.01, *p* = 0.937, *η*^*2*^ = 0.00) or DK/DR responses (*F*(1,61) = 1.34, *p* = 0.252, *η*^*2*^ = 0.02) given for each of the three conditions during the structured interview (accurate, misleading or no gesture). T-tests showed boys were significantly more likely to give an incorrect answer consistent with the misleading gesture than girls *t*(61) = 2.46, *p* = 0.017, Cohen’s *d* = 0.63, indicating that boys were misled by gesture more than girls and that the larger proportion of boys in the older group than the younger group may have affected the results (Fig. 7).

## Discussion

This study examined the capacity of gestural information to mislead, with consideration of how the centrality of a question, or the saliency of a gesture, might impact the gestural misinformation effect. In line with our hypothesis, results showed that misleading gestures were able to corrupt recall of a past event in children between the ages of 5 and 8 years, supporting Kirk et al. (2015) and Broaders and Goldin-Meadow (2010). Contrary to our prediction, and despite research that indicates verbal suggestibility is often a function of age (Bruck & Ceci, 1999; Gudjonsson et al., 2016; Volpini et al., 2016), no age differences were seen between the two age groups, supporting Kirk et al. (2015). Given the gender discrepancies between the two groups, however, and exploratory analysis that demonstrated that girls were less likely to be misled by gesture and were overall more accurate than boys in our sample, the lack of age differences was not conclusive. As predicted, the centrality of a question affected the ability of that question to mislead, with peripheral details showing more susceptibility to gestural misinformation than central events. Also, as predicted, the saliency of a gesture affected the incorporation of misinformation, with salient gestures being more misleading than subtle gestures. These findings show that the gestural misinformation effect is a robust phenomenon, mediated by question centrality and gesture saliency; and demonstrating the forensic importance of these factors for the first time.

Kirk et al. (2015) proposed that the presence of gesture creates a more pervasive false memory effect than speech alone due to stronger encoding during misinformation presentation. It may be that direct activation of the motor system by gesture (Ianì & Bucciarelli, 2017; Wakefield et al., 2013) exploits and reinforces memory for information above that of the original memory even when that gesture is misleading. Our results show that gestural misinformation mislead 47 out of 63 children by at least one misinformation detail, and show support for gestures as a strong communicative tool, even if that gesture contradicts children’s original memory. The idea that false memory increases in line with gist-trace processing and meaningful connections between items (Bouwmeester & Verkoeijen, 2010; Brainerd et al., 2008; Wimmer & Howe, 2009) corresponds with our findings in which the most influential gestures in the current study were the most iconic and accessible for children. Similarly, all gestures were semantically linked to the question asked, i.e., they were not nonsense answers (e.g., when asking what “the man looked like?” the gesture would be plausible such as a ‘hat’ and not nonsensical such as an ‘apple’). Despite this, findings showed that while children were more likely to give an answer consistent with the gesture viewed than any other answer (whether the gesture was accurate or misleading) that this was not universal across all questions or individual participants, indicating individual differences perhaps in children’s prior knowledge base (Bouwmeester & Verkoeijen, 2010; Wimmer & Howe, 2009).

In line with research showing that salient gestures are attended to more by a listener (Chu et al., 2014), making them more obvious as a source of information when a memory trace is weak or when a question is ambiguous (Dargue et al., 2019; Pezdek & Roe, 1995), our results show that salient gestures misled children on more than a third of occasions, with even subtle gestures misleading a tenth of the time. This may be due to the more expressive and visible nature of salient gestures prompting greater neurological engagement than more subtle gestures (Ianì & Bucciarelli, 2017; Yang et al., 2015) or simply that these gestures were the most noticeable as a source of information (Dargue et al., 2019; Pezdek & Roe, 1995). Central information was shown to protect against gestural misinformation with the better integration, improved recall, and stronger memory trace associated with central events (Sarwar et al., 2014), reducing the ability of the gesture to mislead, compared to peripheral details. These findings demonstrate that question centrality and gesture saliency are important considerations during child eyewitness interviews when considering the reliability of evidence.

Further support for the idea that gesture acts as a source of information can be seen in the increased number of questions answered when a gesture was present. This was especially the case for younger children, indicating that the cognitive immaturity of this group, in comparison to older children (Fritz et al., 2010; Geurten & Willems, 2016; Ghetti, 2003), may have affected their recollection of the video when no additional information was present. This can also be seen in younger children’s reduced accuracy for correct answers when compared to the older children, within the control condition.

In addition to the lack of age effects seen between groups, no developmental trend was observed with regard to suggestibility, as argued by Kirk et al. (2015) and despite a wide body of research demonstrating greater suggestibility to *verbal* misinformation in younger children (Bruck & Ceci, 1999; Poole & Dickinson, 2014; Volpini et al., 2016). This was unexpected due to evidence showing the same neurological networks are activated during gesture observation as during speech (Ianì & Bucciarelli, 2017; Yang et al., 2015), indicating a processing similarity such that similar age-related suggestibility effects might be expected. Sampling issues may have affected findings, however, with an uneven gender split between age groups, and the level of familiarity for the video subject (gymnastics) not being formally measured (possibly affecting gist recall/associative activation due to the video being more meaningful to some participants). The narrative skill of participants was not measured in this study, but this may be a useful addition in future studies as there is evidence that advanced narrative abilities can result in greater suggestibility (Kulkofsky & Klemfuss, 2008) and may partially explain reverse developmental trends (Perez et al., 2022). Although evidence here supports a greater narrative ability in girls despite the mean difference in ages in this sample (Boys = 7.07 years, Girls = 6.46 years), it did not follow that this resulted in greater suggestibility, indicating a potential difference between verbal and non-verbal suggestibility. Alternatively, the susceptibility of children to gestural misinformation may *actually* be more resistant to protective cognitive and social developmental advancements, than verbal misinformation; however, given the higher proportion of girls aged 5–6 and boys aged 7–8 years, and exploratory analysis that showed better recall, greater accuracy and reduced gestural suggestibility in girls, conclusions as to developmental effects are limited in the present study and require further research.

An examination of IOIs recalled post-interview showed no information consistent with the misleading gesture given, contrary to previous findings (Broaders & Goldin-Meadow, 2010; Kirk et al., 2015). This may be due to differences in study design. Although Kirk et al. (2015) found that one-third of children included gestural misinformation during post-interview recall, most of the children were a younger age (3-year-olds) than the children in the present study. Between participant designs, with each child assigned to either accurate or misleading conditions and no baseline control, may also have reduced the ability of prior studies to discern whether gestural misinformation would have been present regardless of the gesture used, or was specifically linked to the misleading gesture given. Previous research also found three-quarters of children affirmed at least one untrue suggestion during free recall; however, this finding was drawn from data collapsed across four interview sessions, potentially increasing the chances of this finding (Broaders & Goldin-Meadow, 2010). The focus of the present study to ensure a format more relevant to real-life interviews, including low saliency gestures and a limited number of misleading gestures during interview, may have reduced the power of the study to find a misleading gesture effect during free recall compared to previous studies.

Research by Riesthuis et al. (2022) showed that memory researchers mostly agreed that the SESOI in false memory research should be either any difference leading to *p* < 0.05 (*n* = 7) or any reliable effect size at all (*n* = 2). At least one misinformation detail (*n* = 10) or a raw mean difference of anything up to one misinformation detail (*n* = 4) was also accepted as the SESOI by 14 researchers, while two said they would require two or three misinformation details. In the current study, nearly three-quarters of children were misled by at least one question, with nearly one-third misled by two or more questions, and an average of 1.12 misinformation details. Riesthuis et al. (2022) considered what a small/medium/large effect size is in the misinformation literature. A small effect size was considered to be 0.7 and a medium effect size 1.27, indicating the calculated effect size for misinformation details in this study to be small/medium (0.104). Taking Riesthuis et al.’s (2022) research into consideration, the findings have strong practical and psychological implications for child eyewitness performance due to the negative consequences of any false memory details.

In conclusion, the results from this study support the gestural misinformation effect, with improvements in methodology strengthening the body of work in this area. This study also extends previous findings by demonstrating the importance of question centrality and gesture saliency. The findings have implications for guidelines regarding investigative interviews with children. Interview guidelines need to be updated to include instructions about the impact of gestural information, to help secure the best evidence possible. Investigators should be aware that even subtle gestures may mislead child eyewitnesses, and that evidence regarding peripheral details is vulnerable and prone to disruption. The gestural misinformation effect was shown to be notable regardless of age, indicating that care should be taken when interviewing children of all ages, with additional awareness of possible gender differences. In summary, it is advisable that police interview guidelines are revised to include warnings that gestural information can mislead witnesses, and that appropriate prevention measures are accordingly put in place. This should include video recording all interviews and checking for gestural cues that may have been given wittingly or unwittingly by the interviewer.

## Data Availability

Anonymised data will be made available by the corresponding author upon request.
